# A New Dynamic Response to Therapy Assessment in Postoperative Patients With Low-Risk Differentiated Thyroid Cancer Treated Without Radioactive Iodine

**DOI:** 10.3389/fonc.2021.764258

**Published:** 2021-11-29

**Authors:** Ping Dong, Li Wang, Liu Xiao, Liu Yang, Rui Huang, Lin Li

**Affiliations:** ^1^ Department of Nuclear Medicine, West China Hospital, Sichuan University, Chengdu, China; ^2^ Department of Pancreatic Surgery, West China Hospital, Sichuan University, Chengdu, China

**Keywords:** differentiated thyroid cancer, low-risk, response to therapy assessment, thyroglobulin, neck ultrasonography

## Abstract

**Background:**

Total thyroidectomy (TT) or lobectomy without radioactive iodine (RAI) is becoming a common management for patients with low-risk differentiated thyroid cancer (DTC). However, the assessment of response to therapy for these patients remains controversial. The aim of this study was to propose and validate a new dynamic evaluation strategy to assess the response to therapy in patients with low-risk DTC treated with TT or lobectomy but without RAI.

**Methods:**

We performed a retrospective analysis of 543 adult patients with low-risk DTC who underwent TT or lobectomy without RAI therapy. Follow-up consisted of trends of serum thyroglobulin (Tg), anti-thyroglobulin antibody (TgAb) levels and neck ultrasonography (US) were conducted every 6–24 months. Response to therapy assessments were defined as excellent response, biochemical incomplete response, structural incomplete response, and indeterminate response according to the follow-up findings.

**Results:**

At a median follow-up of 51 months (range 33–66 months), 517 (95%) had excellent response, while the other 26 had either biochemical incomplete response (an increasing trend of suppressed serum Tg levels, n=9; an increasing trend of TgAb levels, n=3) or indeterminate response (a stable or decreasing trend of suppressed serum Tg levels, but a stable positive trend of TgAb levels, n=14). No patients had structural incomplete response or no deaths related to thyroid cancer. The risk of incomplete response was significantly higher in lobectomy than in TT (p<0.001).

**Conclusion:**

Our study proposed and validated a new dynamic response to therapy assessment depending on trends of suppressed serum Tg, TgAb levels, and neck US findings which could be an appropriate tool for postoperative follow-up in low-risk DTC patients without RAI therapy. Our findings provided further evidence to support no routine recommendation of RAI after surgery in low-risk DTC.

## Introduction

The prevalence of low-risk differentiated thyroid cancer (DTC) is increasing significantly, which mainly due to the early diagnosis of thyroid microcarcinoma (TMC) by using neck ultrasonography (US) (1–4). Optimal management of DTC usually requires inter-disciplinary cooperation, including surgery, risk-adapted postoperative radioactive iodine (RAI) therapy, individualized thyroid hormone therapy, and follow-up for the detection of patients with persistent or recurrent disease (5–7). Recently, considering factors such as the excellent prognosis of low-risk DTC (5, 8), absence of significant reduction in recurrence rate or disease-free survival in low-risk patients treated with RAI (9, 10), scarce evidence concerning the usefulness of RAI in improving disease-specific mortality in low-risk DTC (5), and potential side effects on RAI [e.g., chronic sialadenitis (11, 12), secondary malignancies (13, 14)], the 2015 American Thyroid Association (ATA) guidelines recommend performing conservative strategies, namely, total thyroidectomy (TT) or lobectomy without RAI ablation, for low-risk DTC patients (5). Thus, in China, TT or lobectomy without RAI ablation is becoming a common management for patients with low-risk DTC.

Although the above-mentioned conservative strategies are gradually becoming accepted, the assessment of response to therapy for these patients remains controversial. According to the 2015 ATA guidelines, periodic measurements of serum thyroglobulin (Tg) on thyroid hormone therapy and neck US should be considered during the follow-up of patients with low-risk DTC who underwent TT or lobectomy without RAI ablation (5), but the roles played by each of these methods are not specially defined. In 2016, Momesso et al. proposed a dynamic risk stratification method (mainly based on neck US findings and different suppressed serum Tg cutoff values, namely, Tg <0.2, 0.2–5, or >5 ng/mL for TT; Tg <30 or >30 ng/mL for lobectomy, to stratify assessments as excellent response, indeterminate response, biochemical incomplete response and structural incomplete response) to evaluate the response to initial surgery in low-risk DTC patients who did not undergo RAI therapy (15). However, since this evaluation system could be affected by the size of remnant tissue, the results vary significantly [e.g., excellent response, 94.1% as reported by Momesso et al. (15) *versus* 71.7% as reported by Park et al. (16)].

The current study attempted to use a dynamic evaluation strategy based on the trends of suppressed Tg, anti-thyroglobulin antibody (TgAb) levels and neck US findings to determine the response to therapy in Chinese low-risk DTC patients treated with surgery alone (TT or lobectomy) after a median follow-up of 51 months and identify risk factors associated with incomplete response.

## Material and Methods

### Patients

This retrospective study was approved by the Institutional Research Ethics Committee of West China Hospital of Sichuan University (# 20201158). The requirement for written informed consent was waived because this study was of retrospective design and used only de-identified clinicopathologic data.

Electronic medical records at West China Hospital of Sichuan University, Chengdu, China, were retrospectively reviewed for adult patients with low-risk DTC who underwent TT or lobectomy without RAI remnant ablation therapy between July 2015 and September 2016. The inclusion criteria were as follows: patients aged >18 years at the time of surgery; patients with documented low-risk DTC who underwent lobectomy with isthmusectomy or TT, and/or central/lateral neck dissection, without RAI remnant ablation therapy, with thyroid-stimulating hormone (TSH) suppressive therapy; and those who were routinely followed up every 6–24 months with the determination of serum TSH, Tg and TgAb levels and neck US findings. The exclusion criteria were histopathological diagnosis other than DTC, history of other cancers, presence of other conditions that have clinical significance, and absence of sufficient follow-up data.

### Laboratory Studies and Follow-Up Protocol

Serum Tg, TgAb, and TSH levels were measured 1.5–3 months post-operation and during routine follow-up every 6–24 months in our hospital. Between July 2015 and May 2020, serum Tg, TgAb, and TSH levels were measured using a fully automated electrochemiluminescent immunoassay analyzer (Cobas^®^ e 601, Immunoassay Analyzer, Roche, Switzerland) with a measuring range of 0.04–500 ng/mL, 10–4000 IU/mL, and 0.005–100 mIU/L, respectively. After May 2020, serum Tg, TgAb, and TSH levels were measured using a new generation of electrochemiluminescent immunoassay analyzer (Cobas^®^ e 801, Immunoassay Analyzer, Roche, Switzerland). Neck US examination was performed by experienced operators, using color Doppler scanners with multi-frequency probes (7.5–10 MHz), every 6 months during the first year post-operation and repeated at 12- to 24- month intervals thereafter.

The trends of suppressed serum Tg and TgAb levels were evaluated at the same TSH levels and defined as stable (the change of Tg or TgAb levels <20% when comparing the three consecutive Tg or TgAb levels), decreasing (the decrease of Tg or TgAb levels ≥20%) or increasing (the increase of Tg or TgAb levels ≥20%). Positive TgAb was defined as serum TgAb level ≥60 IU/mL, which might interfere with Tg measurement (16, 17). When the serum TgAb level was <60 IU/mL, serum TgAb status was defined as negative (16).

Neck US examination included the analysis of the thyroid bed, remnant thyroid tissue, and lymph node regions. A negative neck US result was defined as an empty thyroid bed with the jugular and carotid vessels in a medial location or no abnormalities in the remnant thyroid tissue, and as the absence of suspicious lymph nodes (LNs).

### Response to Therapy Assessments

Response to therapy assessments at the last follow-up were defined as excellent response, biochemical incomplete response, structural incomplete response, and indeterminate response, which mainly depended on the trends of serum suppressed Tg, TgAb levels and neck US findings with avoidance of the size of remnant tissue. The definitions of each response to therapy assessment at the last follow-up for patients who underwent TT or lobectomy without RAI are shown in **Table 1** (15, 16, 18, 19).

**Table 1:** The definitions of a new response to therapy assessment in patients with low-risk DTC who underwent total thyroidectomy or lobectomy without RAI.

	Definitions
Excellent response	Negative neck US^a^ and a stable or decreasing trend of suppressed serum Tg^b^ and negative TgAb^c^ levels
Biochemical incomplete response	Negative neck US and an increasing trend of suppressed serum Tg or TgAb levels
Structural incomplete response	Structural evidence of disease regardless of serum Tg or TgAb levels
Indeterminate response	A stable or decreasing trend of suppressed serum Tg levels, and nonspecific neck US findings or a stable positive trend of TgAb levels

DTC, differentiated thyroid cancer; RAI, radioactive iodine; US, ultrasonography; TgAb, anti-thyroglobulin antibody; Tg, thyroglobulin.

a A negative neck US result was defined as an empty thyroid bed with the jugular and carotid vessels in a medial location or no abnormalities in the remnant thyroid tissue, and as the absence of suspicious lymph nodes.

b Trend of suppressed serum Tg was evaluated at the similar thyrotropin levels and defined as: stable (the change of Tg levels <20% when comparing the three consecutive Tg levels), decreasing (the decrease of Tg levels ≥20%) or increasing (the increase of Tg levels ≥20%).

c Positive serum TgAb was defined as TgAb level ≥60 IU/mL; negative TgAb was defined as <60 IU/mL. The trends in the change of TgAb levels were defined as stable (the change of TgAb levels <20% when comparing the three consecutive TgAb levels), decreasing (the decrease of TgAb levels ≥20%), or increasing (the increase of TgAb levels ≥20%).

### Statistical Analysis

Continuous variables were presented as means and standard deviations or median values with ranges, and categorical variables were calculated as frequencies or percentages. A comparison of continuous variables was performed with Student’s t-test, and that of categorical variables was performed using Pearson’s χ^2^ test or Fisher’s exact test. Prognostic factors associated with incomplete response at the last follow-up were analyzed using logistic regression. A p-value of <0.05 was considered statistically significant. Statistical analyses were performed using IBM SPSS statistical software (version 23.0 for Mac OS X).

## Results

### Study Cohort

**Figure 1:**
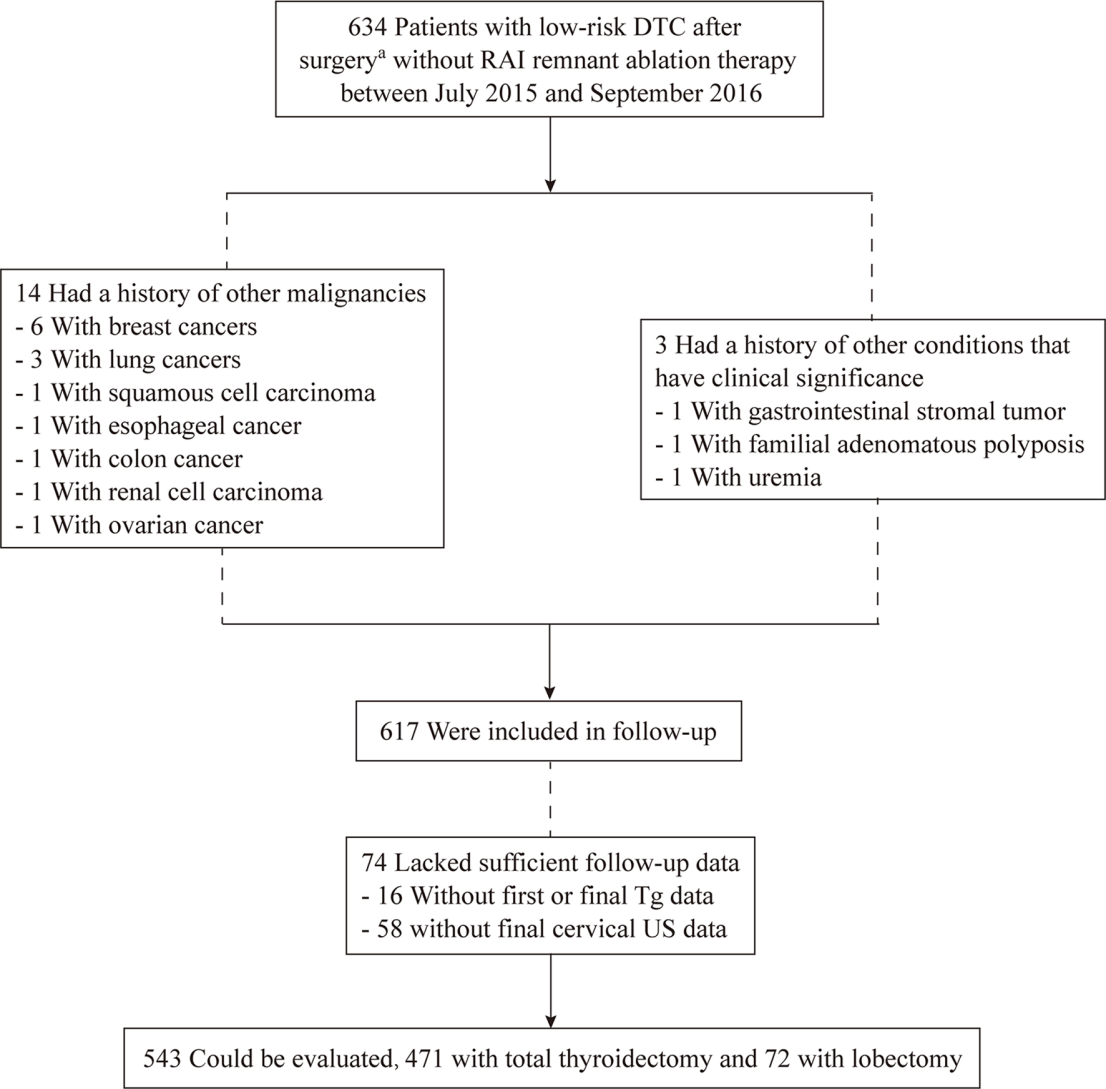
Flow chart of inclusion-exclusion of patients with low-risk differentiated thyroid cancer without radioiodine ablation at the study. ^a^Patients underwent lobectomy + isthmusectomy + ipsilateral central neck dissection or total thyroidectomy +/- central/lateral neck dissection.

Between July 2015 and September 2016, a total of 634 patients were included (**Figure 1**). Seventeen patients were not included in the follow-up: 14 had other cancers (six with breast cancer, three with lung cancer, one with squamous cell carcinoma, one with esophageal cancer, one with colon cancer, one with renal cell carcinoma, and one with ovarian cancer), while 3 had a history of other conditions that had clinical significance (one with gastrointestinal stromal tumor, one with familial adenomatous polyposis, and one with uremia). Seventy-four patients could not be evaluated because of insufficient follow-up data. Finally, 543 patients were evaluated: 471 (87%) who underwent TT and 72 (13%) who underwent lobectomy.

### Clinical Characteristics

The demographic characteristics and clinical features of the 543 patients included in the study are shown in **Table 2**. Mean patient age was 43 years; 79% were female, and all patients had low-risk papillary thyroid cancers. TMC was found in 420 (77%) of the 543 patients, wherein 19% had multifocal disease. Neck dissection was performed in 540 (99%) of the 543 patients, wherein 77% had N0. Central cervical LN metastases were found in 125 (23%) of the 543 patients, including 73 (58%) with one LN metastasis, 34 (27%) with two LN metastases, and 18 (15%) with three or four LN metastases. No patients had lateral LN cervical metastases. Based on the 8^th^ American Joint Cancer Committee TNM staging system, 97% and 3% patients had stage I and stage II, respectively. For thyroid surgery specific complications, rates of transient hypoparathyroidism and vocal cord paralysis in TT were higher than those in lobectomy.

**Table 2:** Baseline characteristics of the patients.

	Entire Cohort (n = 543)	Surgery		p
		Total thyroidectomy^b^(n = 471)	Lobectomy^c^(n = 72)	
Age (years), median (range)	43 (19–79)	44 (19–79)	40 (19–60)	0.014
Sex, no. of patients (%)				0.046
Male	116 (21)	94 (20)	22 (31)	
Female	427 (79)	377 (80)	50 (69)	
Size of largest focus (mm), mean (range)	8.6 (1.5–29)	8.8 (1.5–29)	7.4 (4–15)	0.009
Focality, no. of patients (%)				0.005
Unifocal	440 (81)	373 (79)	67 (93)	
Multifocal	103 (19)	98 (21)	5 (7)	0.927
2 foci	84 (81)	80 (82)	4 (80)	
3–5 foci	19 (19)	18 (18)	1 (20)	
T classification^a^, no. of patients (%)				0.013
T1a	420 (77)	355 (75)	65 (90)	
T1b	108 (20)	101 (21)	7 (10)	
T2	15 (3)	15 (3)	0 (0)	
N classification^a^, no. of patients (%)				0.228
N0	418 (77)	367 (78)	51 (71)	
N1a	125 (23)	104 (22)	21 (29)	0.839
1 LN	73 (58)	59 (57)	14 (67)	
2 LN	34 (27)	29 (28)	5 (24)	
3–4 LN	18 (15)	16 (15)	2 (9)	
TNM Staging^a^, no. of patients (%)				0.708
Stage I	527 (97)	456 (97)	71 (99)	
Stage II	16 (3)	15 (3)	1 (1)	
Follow-up duration (months), mean (range)	49 (31–64)	49 (32–64)	47 (31–62)	0.021

T, tumor; N, node; LN, lymph node; M, metastasis.

a TNM staging is determined by eighth American Joint Cancer Committee TNM staging system.

b There were 3 patients underwent total thyroidectomy, 129 patients underwent total thyroidectomy + ipsilateral central neck dissection, 334 patients underwent total thyroidectomy + bilateral central neck dissection, 5 patients underwent total thyroidectomy + bilateral central and lateral neck dissection.

c There were 72 patients underwent lobectomy + isthmusectomy + ipsilateral central neck dissection.

Patients who underwent lobectomy were younger than those who underwent TT (p=0.014). More unifocal disease and TMCs were found in patients who underwent lobectomy than in those who underwent TT (p=0.005 and p=0.013, respectively). There were no significant differences in LN metastasis or TNM staging between the TT and lobectomy cohorts (**Table 2**).

### Response to Therapy Assessments

At the last follow-up (median follow-up, 51 months; range, 33–66 months), 517 (95%) of the 543 patients had excellent response (**Table 3**). Of the other 26 patients, 12 had biochemical incomplete response (an increasing trend of suppressed serum Tg levels, n=9; an increasing trend of TgAb levels, n=3), 14 had indeterminate response (a stable or decreasing trend of suppressed serum Tg levels, but a stable positive trend of TgAb levels). No patients had structural incomplete response or no deaths related to thyroid cancer.

**Table 3:** Response to therapy assessments at the last follow-up in the study subjects.

Response to therapy assessments^a^	Total (n = 543)	Total thyroidectomy (n = 471)	Lobectomy (n = 72)	p
Excellent response %	517 (95)	457 (97)	60 (83)	<0.001
Biochemical incomplete response %	12 (2)	3 (1)	9 (13)	<0.001
Indeterminate incomplete response %	14 (3)	11 (2)	3 (4)	0.413

a There were no patients with structural incomplete response.

Excellent response and fewer biochemical incomplete response were found in patients who underwent TT than in those who underwent lobectomy (p<0.001). There were no significant differences in the indeterminate incomplete response between the two groups (**Table 3**).

### Prognostic Factors Associated With Incomplete Response

Details and the results of univariate analysis of prognostic factors associated with incomplete response in low-risk DTC patients treated with TT or lobectomy without RAI therapy at the last follow-up are presented in **Table 4**. The risk of incomplete response at the last follow-up was not related to the patient’s age, sex, primary tumor, or nodal status (**Table 4**). The risk of incomplete response was significantly higher in patients who underwent lobectomy than their counterpart (odds ratio=6.529, p=0.001).

**Table 4:** Univariate analysis of prognostic factors associated with incomplete response at the last follow-up in 543 patients.

	No. of incomplete responses/no. of patients (%)	Odds ratio	95% CI	p
Age (years)				
≤55	24/458 (5.2)	1 (ref)		
>55	2/85 (2.4)	2.295	0.532–9.897	0.265
Sex				
Male	5/116 (4.3)	1 (ref)		
Female	21/427 (4.9)	0.871	0.321–2.362	0.786
Surgery				
Lobectomy	12/72 (16.7)	1 (ref)		
Total thyroidectomy	14/471 (3.0)	6.529	2.885–14.774	<0.001
Focality				
Unifocal	22/440 (5.0)	1 (ref)		
Multifocal	4/103 (3.9)	1.303	0.439–3.865	0.634
T classification^a^				
T1a	22/420 (5.2)	1 (ref)		
T1b/T2	4/123 (3.3)	1.644	0.556–4.866	0.369
N classification^a^				
N0	19/418 (4.5)	1 (ref)		
N1a	7/125 (5.6)	0.803	0.329–1.956	0.629

T, tumor; N, node.

a TNM staging is determined by eighth American Joint Cancer Committee TNM staging system.

## Discussion

In this study, we proposed and validated a new dynamic response to therapy assessment depending on trends of suppressed serum Tg, TgAb levels, and neck US findings in patients with low-risk DTC who underwent TT or lobectomy without RAI therapy. Response to therapy assessments were defined as excellent response, biochemical incomplete response, structural incomplete response, and indeterminate response, which mainly depended on the trends of suppressed serum Tg, TgAb levels and neck US findings without interference of the size of remnant tissue. Our findings show that, at the last follow-up, 95% of patients with low-risk DTC who underwent TT or lobectomy without RAI ablation therapy had excellent response. This rate in our cohort was similar to that (94.1%) reported by Momesso et al. (15), thus conforming the definition used in our study might be reasonable and reliable. Furthermore, the proportion of patients (97%) with excellent response among those who underwent TT in our study was similar to the proportion (98%) reported by Schlumberger et al. who performed a 5-year follow-up study of a randomized, phase 3, equivalence trial (ESTIMABL1, two thyrotropin-stimulation methods: thyroid hormone withdrawal *versus* the use of recombinant human TSH, and two RAI ablation doses:1.1 GBq *versus* 3.7 GBq) (20). Thus, our study provided further evidence to support no routine recommendation of RAI after surgery in low-risk DTC. The proportion of patients with biochemical incomplete response or indeterminate response in our study was in accordance with the 10-year recurrence rate of 1%–2%, which is expected for patients with low-risk DTC. During our follow-up, no patients had structural incomplete response, which may be explained by the aggressive prophylactic lymph node dissection and insufficient follow-up duration (21, 22).

According to the 2015 ATA guidelines, lobectomy may be sufficient for a unifocal intrathyroidal low-risk carcinoma sized <4 cm in diameter in patients with no prior head and neck radiation, familial thyroid cancer, or clinically detectable cervical LN metastases (5). In the present study, type of surgery was found to be a prognostic factor associated with incomplete response in low-risk DTC patients without RAI therapy at the last follow-up. Particularly, we found that the risk of biochemical incomplete response was significantly higher in patients who underwent lobectomy (seven and two patients due to increasing trends of suppressed serum Tg and TgAb levels, respectively), when compared to TT (two and one patients due to an increasing trend of suppressed serum Tg and TgAb levels, respectively). For low-risk DTC patients treated with lobectomy, an increase in Tg values over time suggested a growing thyroid tissue or tumor, and an increase in TgAb values suggested coexistent Hashimoto thyroiditis in residual thyroid tissues (especially in patients diagnosed with Hashimoto thyroiditis by surgical histopathology) or tumor relapse (5, 23–25).

The present study has several strengths. First, postoperative risk assessment, assessment of potential benefits and side effects of RAI therapy, and patients’ preferences and values had been adequately considered during post-operative management (observation and individualized thyroid hormone therapy without RAI ablation). Second, all the included patients underwent TT or lobectomy by experienced thyroid surgeons in our hospital who had more than 10 years of thyroidectomy experience. Third, all the serum Tg, TgAb, and TSH levels of all patients were measured by electrochemiluminescent immunoassay in our laboratory to ensure the accuracy and reliability. Finally, considering that neck US is operator-dependent, all high-resolution neck US scans were performed by experienced specialists to evaluate structural abnormalities to ensure the accuracy in our center. However, this study has several limitations. First, the 51-month (median) follow-up period chosen for this study might be suboptimal. Although most recurrences (80%) in low-risk DTC patients occurred during the first 3-5 years of follow-up, some recurrence or incomplete response might be missed (26, 27). Second, the single-institutional study design creates selection biases that are difficult to control. Third, this retrospective study enrolled a relatively small cohort of patients who underwent lobectomy.

In conclusion, our study validates that the newly proposed dynamic response to therapy assessment depending on trends of suppressed serum Tg, TgAb levels, and neck US findings could be an appropriate tool for postoperative follow-up in low-risk DTC patients without RAI therapy. Our findings provide further evidence to support no routine recommendation of RAI after surgery in low-risk DTC.

## Data Availability Statement

The original contributions presented in the study are included in the article/supplementary material. Further inquiries can be directed to the corresponding authors.

## Ethics Statement

The studies involving human participants were reviewed and approved by the Institutional Research Ethics Committee of West China Hospital of Sichuan University. Written informed consent for participation was not required for this study in accordance with the national legislation and the institutional requirements.

## Author Contributions

PD, LW, RH and LL designed this research. PD, LW, LX and LY collected the data and performed the statistical analyses. PD, LW and RH reviewed the results, interpreted the data, and wrote the manuscript. PD, LW, RH and LL discussed and edited the paper. All authors contributed to the article and approved the submitted version.

## Funding

This study was supported by the 1.3.5 Project for Disciplines of Excellence, West China Hospital, Sichuan University (No. ZYGD18016).

## Conflict of Interest

The authors declare that the research was conducted in the absence of any commercial or financial relationships that could be construed as a potential conflict of interest.

## Publisher’s Note

All claims expressed in this article are solely those of the authors and do not necessarily represent those of their affiliated organizations, or those of the publisher, the editors and the reviewers. Any product that may be evaluated in this article, or claim that may be made by its manufacturer, is not guaranteed or endorsed by the publisher.
